# Reverse Dorsoradial Digital Artery Flap for Reconstruction of Thumb Soft Tissue Defects: A Prospective Clinical Series

**DOI:** 10.1016/j.jhsg.2026.101107

**Published:** 2026-08-07

**Authors:** Cong-Dien Duong, Quoc-Khanh Pham-Trinh, Phuc-Nguyen Huynh, Van-Duong Tran, Anh-Tan Tran, Ngoc Hoang-Van

**Affiliations:** ∗ Department of Plastic, Reconstructive and Burn Surgery, Can Tho Central General Hospital, Can Tho, Vietnam; † Department of Plastic, Reconstructive and Aesthetic Surgery, Can Tho University of Medicine and Pharmacy, Can Tho, Vietnam; ‡ Can Tho University of Medicine and Pharmacy, Can Tho, Vietnam; § Department of Plastic and Aesthetic Microsurgery, Cho Ray Hospital, Ho Chi Minh City, Vietnam; ‖ Unit of Aesthetic and Plastic Surgery, Binh Chanh General Hospital, Ho Chi Minh City, Vietnam

**Keywords:** Dorsoradial digital artery flap, Hand surgery, Homodigital flap, Soft tissue defect, Thumb reconstruction

## Abstract

**Purpose:**

Soft tissue defects of the thumb exposing tendon or bone require durable, sensate, single-stage coverage. The reverse homodigital dorsoradial digital artery flap is supplied by a constant axial vessel from the radial artery. This prospective case series evaluated its outcomes and identified factors associated with motion, sensory, and scar recovery.

**Methods:**

Seventeen consecutive patients aged ≥16 years with thumb defects exposing tendon or bone were treated with this flap at a single tertiary referral center between June 2024 and December 2025. Thumb motion was graded with the American Society for Surgery of the Hand criteria, sensation with the American Society of Hand Therapists scale, and scar with the Vancouver Scar Scale at 3 months.

**Results:**

Mean age was 41.3 ± 17.0 years (range, 17–69); 88.2% were men. Defects involved the pulp (47.1%), volar (29.4%), or dorsal (23.5%) aspect; mean flap area was 8.5 ± 2.5 cm^2^ (range, 4–12 cm^2^). All flaps survived completely (100%); donor sites were closed primarily in 12 (70.6%) and skin grafted in 5 (29.4%). At 3 months, the American Society for Surgery of the Hand motion was good in 14 patients (82.4%), the American Society of Hand Therapists sensation reached S3 in 12 (70.6%), and the mean Vancouver Scar Scale score was 2.94 ± 1.02. Larger flap area was associated with poorer sensory recovery, and crush/avulsion injuries with higher Vancouver Scar Scale scores.

**Conclusions:**

The reverse dorsoradial digital artery flap is a safe, reliable, single-stage option for thumb soft tissue defects with high survival, satisfactory motion and sensation, and minimal donor site morbidity. The absence of a control group precludes establishing superiority over alternative reconstructive options.

**Type of study/level of evidence:**

Therapeutic IV.

The thumb contributes approximately 50% of overall hand function and is indispensable for opposition, pinch, and grasp.^1^ Because of its central role in nearly every prehensile task, the thumb is highly exposed to occupational and domestic injury, and even modest soft tissue loss may critically impair fine motor performance. The volar surface, in particular, depends on glabrous, sensate, and durable skin to allow precise tactile feedback during prehension; the digital nerves, vessels, and tendons lie immediately beneath this thin coverage.

The principles of thumb soft tissue reconstruction extend beyond simple wound closure: ideal management aims to preserve length, restore sensation, recover range of motion (ROM), and achieve an acceptable cosmetic outcome.^2^^,^^3^ When tendon, bone, or joint surfaces are exposed, skin grafting alone is unreliable, and flap coverage becomes mandatory.^4^ A wide spectrum of options has been described, including local advancement and rotation flaps, regional pedicled flaps such as the first dorsal metacarpal artery (FDMA, or Foucher) flap, crossfinger and Littler flaps, and free tissue transfer.^5^, ^6^, ^7^, ^8^

Among these, the reverse homodigital dorsoradial digital artery flap—commonly referred to as the Moschella flap—has gained increasing acceptance as a versatile, single-stage solution for distal thumb defects. The flap is harvested from the dorsoradial aspect of the proximal phalanx and metacarpal of the thumb and is based on a constant axial vessel arising from the radial artery, with a reliable distal anastomosis to the palmar vascular network at approximately the middle third of the proximal phalanx.^4^^,^^9^ The reverse dorsoradial flap is an axial pattern arterial flap supplied by the dorsoradial digital artery in reverse direction, with venous drainage through paired venae comitantes. It is not a venous flap of the Yoshimura type, and the venous congestion-related limitations of pure venous flaps therefore do not apply. Its principal advantages include thin and pliable, nearly hairless skin; consistent vascular anatomy; preservation of the major digital arteries; minimal donor site morbidity; and the ability to cover defects extending to the thumb tip in a single surgery while immobilizing only the injured digit.^9^^,^^10^ The principal regional alternative is the FDMA (Foucher, or “kite”) flap, recently re-illustrated by Zyluk and Flicinski^11^ in a contemporary technique-focused series, which remains the most widely used pedicled option but requires proximal dissection and immobilization of an uninjured digit.

Despite the growing literature, prospective clinical series specifically evaluating the reverse dorsoradial digital artery flap in lower- and middle-income surgical settings remain limited, and factors influencing functional and aesthetic recovery are incompletely characterized. The purpose of this study was to evaluate the clinical outcomes of thumb soft tissue reconstruction using the reverse dorsoradial digital artery flap and to analyze defect- and technique-related factors associated with motion, sensory, and scar outcomes.

## Materials and Methods

### Study design and ethics

We conducted a prospective, descriptive, single-center case series at a tertiary referral hospital between June 2024 and December 2025. The study was approved by the Biomedical Research Ethics Committee of the affiliated medical university (approval No. 24.352.HV-DHYDCT, dated June 28, 2024) and was conducted in accordance with the Declaration of Helsinki. Written informed consent was obtained from each patient before surgery and again before publication of any clinical photographs.

Because the study does not involve prospective assignment of participants to an intervention or comparison group (all patients received the same reconstructive procedure as part of routine clinical care), it does not meet the four International Committee of Medical Journal Editors criteria for a clinical trial and was therefore not prospectively registered in a clinical trials registry. This is a level IV therapeutic case series, and the Consolidated Standards Of Reporting Trials guidelines, which apply to randomized controlled trials, are not applicable to this study design.

### Patients

Consecutive patients aged 16 years or older presenting with a soft tissue defect of the thumb that exposed underlying tendon or bone and was deemed amenable to coverage by a reverse dorsoradial digital artery flap were eligible. Exclusion criteria were degloving injury of the entire thumb, concomitant injury to the donor site or to both digital arteries of the thumb, underlying obliterative arteriopathy or peripheral vascular disease, and any documented coagulopathy. All patients consented to participate.

### Variables and outcome measures

Recorded variables included age, sex, comorbidities, mechanism of injury, time from injury to coverage, defect location and size, associated tendon and/or bone injury, contamination grade (clean, contaminated, or infected), wound character (sharp, irregular, or crush/avulsion), and intraoperative technical details (flap dimensions, donor site management, pedicle length, fixation).

Early outcomes (within 14 postoperative days) included completeness of defect coverage, flap survival (complete survival; partial necrosis < 1/3 of the flap; or total necrosis), and donor site healing. Late outcomes were assessed at 3 months. Specifically, thumb motion was graded using the criteria of the American Society for Surgery of the Hand (ASSH) as excellent, good, fair, or poor. Sensation was assessed with the American Society of Hand Therapists scale (S0 to S4), incorporating Semmes–Weinstein monofilament testing and static two-point discrimination.^12^ Scar quality was rated with the Vancouver Scar Scale (VSS, 0–13). Patient satisfaction was rated on a 5-point Likert scale (very dissatisfied to very satisfied).

### Surgical technique

The surgery was performed in nine reproducible steps under axillary brachial plexus block with arm tourniquet control, after radical wound debridement and copious lavage of the recipient defect:

1. Preoperative marking: The recipient defect dimensions were measured, and the planned flap was outlined on the dorsoradial aspect of the thumb metacarpal and proximal phalanx, designed approximately 20% larger than the defect to allow tension-free inset (Fig. A).

**Figure:**
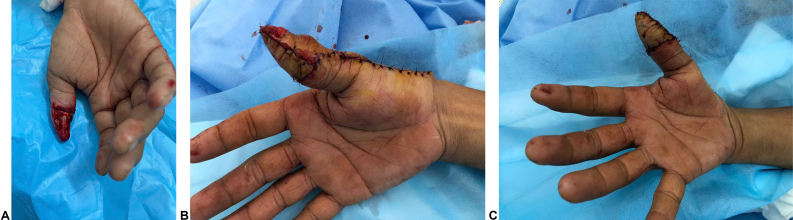
Surgical sequence of the reverse dorsoradial digital artery flap of the thumb. **A** Preoperative soft tissue defect of the thumb pulp with exposure of the distal phalanx. **B** Immediate postoperative appearance after rotation and inset of the reverse dorsoradial digital artery flap, with primary closure of the donor site and visible suture line along the dorsoradial border of the thumb. The dusky discoloration visible in panel **B** represents physiological venous reactive hyperemia immediately after tourniquet release together with povidone-iodine surgical skin staining; it resolved within 48 hours without intervention, and no flap developed partial necrosis or epidermolysis. **C** Postoperative dorsoradial view of the right hand demonstrating complete coverage of the thumb defect; the flap color, contour, and the position of adjacent fingers are preserved.
2. Pivot point identification: The pivot point was marked at the midpoint of the proximal phalanx, where the dorsoradial digital artery anastomoses with the palmar vascular network. The pedicle was designed 3–4 mm longer than the linear pivot-to-defect distance to avoid kinking or torsion after flap rotation.
3. Skin incision: A sharp circumferential skin incision was made at the distal margin of the flap.
4. Flap elevation: The flap was elevated immediately superficial to the extensor pollicis brevis paratenon, proceeding proximal-to-distal, including a generous cuff of subcutaneous fat over the pedicle to protect the axial vessel and ensure venous drainage. The artery was not separately skeletonized.
5. Management of the dorsal sensory branch of the radial nerve (DSBRN): Encountered branches were sharply divided. The proximal stump was buried away from the wound bed to prevent painful neuroma; the distal segment was incorporated into the flap when sensation transfer was desired.
6. Pedicle dissection: The pedicle was approached through a lazy-S or zigzag dorsal incision, raised in continuity with the flap, and dissected toward the pivot point with careful preservation of the palmar-network anastomosis.
7. Tourniquet release and perfusion confirmation: After release, capillary refill and dermal bleeding at the distal flap edge were confirmed before inset.
8. Flap inset: The flap was rotated and inset into the defect with loose, tension-free interrupted sutures (Fig. B).
9. Donor site closure: Defects ≤ 10 cm^2^ were closed primarily; larger defects were resurfaced with a full-thickness skin graft harvested from the volar wrist crease (Fig. C).^13^

After surgery, the thumb was immobilized in a thermoplastic splint for 14 days, after which active ROM therapy was initiated under hand therapist supervision. When concomitant fractures were present, a Kirschner wire was placed and removed at 3 weeks, after which mobilization was progressed.

### Statistical analysis

Data were recorded on standardized case report forms and analyzed with SPSS Statistics version 26.0 (IBM Corp). Continuous variables are presented as mean ± standard deviation (range), and categorical variables as frequency and percentage. Given the modest sample size, between-group comparisons of the VSS score across wound characters and of flap area between sensory recovery groups were performed using nonparametric tests (Kruskal–Wallis and Mann–Whitney U), with two-sided *P* < .05 considered statistically significant.

## Results

### Patient and defect characteristics

Seventeen consecutive patients were included. The mean age was 41.3 ± 17.0 years (range, 17–69), and 15 patients (88.2%) were men. The dominant mechanism of injury was machinery-related trauma (cutting, pressing, or sawing devices) in 15 patients (88.2%) and burns in 2 patients (11.8%); injuries were nearly equally distributed between hands. Defects most frequently involved the pulp (47.1%), followed by the volar (29.4%) and dorsal (23.5%) surfaces. Tendon exposure or injury was present in six patients (35.3%), and bony involvement was present in one patient (5.9%). Contamination was present in 10 wounds (58.8%). Seven wounds (41.2%) were caused by crushing or avulsion forces (Table 1).

**Table 1:** Patient Demographics, Defect Characteristics, and Injury Patterns (N = 17).∗

Variable	Category	n	%
Sex	M	15	88.2
	F	2	11.8
Mechanism	Mechanical trauma	15	88.2
	Burn	2	11.8
Side	L	9	52.9
	R	8	47.1
Defect location	Pulp	8	47.1
	Volar	5	29.4
	Dorsal	4	23.5
Associated injury	Tendon exposure/injury	6	35.3
	Bone exposure/fracture	1	5.9
Contamination	Clean	7	41.2
	Contaminated	10	58.8
Wound character	Sharp	5	29.4
	Irregular	5	29.4
	Crush/avulsion	7	41.2

∗ Values are presented as frequency (n) and percentage (%). Percentages may not sum to 100 because of rounding.

### Intraoperative findings and donor site management

All flaps were elevated as planned over the dorsoradial aspect of the proximal phalanx and metacarpal of the thumb, based on the constant axial vessel arising from the radial artery. The mean flap area was 8.5 ± 2.5 cm^2^ (range, 4–12 cm^2^). Twelve donor sites (70.6%) were closed primarily with tension-relieving sutures, and five (29.4%)—all involving flaps larger than 10 cm^2^—required full-thickness skin grafting. All donor sites healed by primary intention with no graft loss, infection, hematoma, or persistent dysesthesia at the donor area.

### Early outcomes

Coverage of the recipient defect was complete in all cases (100%), and flap survival was 100%, with no episode of venous congestion, partial flap necrosis, infection, or postoperative hemorrhage. No flap required surgical revision or secondary procedures during the immediate postoperative period.

### Functional, sensory, and aesthetic outcomes at 3 months

At the 3-month follow-up, ASSH grading of thumb motion was good in 14 patients (82.4%) and fair in three (17.6%). None were graded poor. The American Society of Hand Therapists sensory recovery reached S3 in 12 patients (70.6%) and S2+ in the remaining 5 (29.4%). The mean VSS score was 2.94 ± 1.02 (range, 1–5), and all patients (100%) reported being satisfied or very satisfied with the outcome (Table 2).

**Table 2:** Early and 3-Month Clinical Outcomes After Reverse Dorsoradial Digital Artery Flap (N = 17).

Outcome	Category	n	%
Flap survival	Complete	17	100
Donor site closure	Primary closure	12	70.6
	Skin graft	5	29.4
ASSH motion (3 mo)	Good	14	82.4
	Fair	3	17.6
ASHT sensation (3 mo)∗	S3	12	70.6
	S2+	5	29.4
Patient satisfaction	Satisfied/very satisfied	17	100

ASHT, American Society of Hand Therapists.

∗ Sensory grades follow the ASHT scale: S2+ indicates protective sensation with some discrimination; S3 indicates discriminative sensation with two-point discrimination ≤ 15 mm.

### Factors associated with outcomes

All three patients with a fair ASSH motion grade had concomitant extensor tendon involvement; one of these also had a distal phalanx fracture managed with Kirschner wire fixation removed at 3 weeks before progressive rehabilitation. Diabetes mellitus, present in a small subset of patients, was not associated with worse outcomes when glycemic control was maintained perioperatively.

Wound character was associated with scar quality. Mean VSS scores were 1.80 for sharp, 3.00 for irregular, and 3.71 for crush/avulsion injuries (Table 3). A larger flap area was associated with poorer sensory recovery. Patients with S2+ outcomes had a mean flap area of 11.7 cm^2^ compared with 7.5 cm^2^ in those reaching S3. All five patients requiring donor site skin grafting had flaps exceeding 10 cm^2^. No patient developed long-term donor site pain, dysesthesia, or stiffness in adjacent uninjured digits.

**Table 3:** Mean VSS Score by Wound Character at 3 Months.

Wound character	n	Mean VSS Score∗
Sharp	5	1.80
Irregular	5	3.00
Crush/avulsion	7	3.71

∗ VSS scores range from 0 (normal skin) to 13 (worst possible scar). Higher scores indicate poorer scar quality.

## Discussion

This prospective single-center series demonstrates that the reverse homodigital dorsoradial digital artery flap is a safe, reproducible, single-stage option for thumb soft tissue defects exposing tendon or bone. We observed 100% flap survival, 82.4% good ASSH motion, S3 sensory recovery in more than two-thirds of patients, and a mean VSS of 2.94 with universal patient satisfaction. These results align with the original description by Moschella and Cordova,^4^ who reported reliable coverage and acceptable cosmetic and sensory outcomes in 16 patients, including a mean static two-point discrimination of 9 mm without nerve coaptation. The anatomic basis of the flap—a constant dorsoradial axial vessel arising from the radial artery and anastomosing with the palmar network at approximately the middle third of the proximal phalanx—has been confirmed in cadaveric dissections of 25 thumbs and underpins the consistency of our clinical results.^14^ A recent systematic review of reverse-flow homodigital flaps for the thumb similarly highlighted the robustness of this technique across a heterogeneous body of literature.^9^

The thumb’s functional dominance and exposed position make even moderate-sized soft tissue losses disproportionately disabling.^1^ When tendon, bone, or joint surfaces are exposed, skin grafting is unreliable and durable flap coverage is required.^2^ Compared with regional alternatives, the reverse dorsoradial flap offers several practical advantages. The FDMA (Foucher or kite) flap, recently re-illustrated by Zyluk and Flicinski^11^, and its variants—although versatile—require proximal dissection, may require harvesting of index dorsal skin, and immobilize a noninjured digit; crossfinger and Littler flaps necessitate two-stage division, with adjacent finger immobilization that risks stiffness, particularly in older patients; free tissue transfer demands microsurgical resources that are not universally available.^5^, ^6^, ^7^, ^8^ The reverse homodigital dorsoradial flap, by contrast, can be elevated within the same surgical field, immobilizes only the injured thumb, and exploits glabrous, almost hairless skin that closely matches the recipient site.^4^^,^^10^

Our 100% flap survival rate is consistent with—or slightly better than—previously reported series. Goh et al^9^ in their 2024 systematic review pooling outcomes from multiple cohorts, reported a high overall survival rate for reverse-flow homodigital thumb flaps, and Zhang et al^10^ on long-term follow-up, demonstrated sustained sensory recovery and patient-reported function. The reliability of the technique has been further confirmed in cohort studies from East Asia, including a 42-patient series by Qin et al^15^ that compared narrow versus wide pedicle widths and identified the narrow pedicle (≤0.8 cm) as associated with shorter time to return to work and improved aesthetic satisfaction. We attribute our complete survival rate to several technical points: harvesting a generous adipofascial cuff around the pedicle rather than skeletonizing the artery; preserving the palmar anastomosis at the pivot point; designing the pedicle 3–4 mm longer than the linear pivot-to-defect distance to avoid kinking; and routinely confirming perfusion after tourniquet release before insetting.

Sensory recovery in our cohort was meaningful but not uniform. Twelve of 17 patients (70.6%) reached protective and discriminative sensation at the S3 level by three months despite the flap being primarily an extrinsic (non-innervated by the proper digital nerve of the recipient) reconstruction. We observed that larger flap area correlated with poorer sensory recovery (mean area 11.7 cm^2^ for S2+ vs 7.5 cm^2^ for S3). This finding is biologically plausible, and three mechanisms are likely to contribute. First, the DSBRN provides only a fixed innervation territory; once the flap exceeds the cutaneous area supplied by the nerve branches captured within the pedicle, the peripheral zones of the flap remain dependent on slow collateral reinnervation from the wound bed. Second, collateral neurite ingrowth from recipient-bed axons must traverse a greater distance to reach the geometric center of a larger flap, prolonging the time to reach the S3 threshold of two-point discrimination. Third, in larger flaps reverse perfusion through the distal palmar anastomosis is most attenuated at the geometric center, and a relative metabolic disadvantage in this zone may slow nerve regeneration even when gross flap survival is preserved. Bao et al^16^ demonstrated that intentionally including a branch of the DSBRN within the pedicle and coapting it to a proper digital nerve at the recipient site improved sensory recovery in 7 of 8 patients in their series. Based on our findings and the work of Bao et al, we recommend considering an innervated variant—performed by intentionally including a branch of the DSBRN within the pedicle and coapting it to a recipient proper digital nerve—in three clinical scenarios: (1) anticipated flap dimensions exceeding 10 cm^2^; (2) pulp resurfacing in patients younger than 40 years; and (3) any patient in a manually demanding occupation in whom precise two-point discrimination is critical to return to work. In smaller defects (<8 cm^2^) on volar or dorsal nonpulp surfaces, the noninnervated dorsoradial flap appears to provide adequate sensory recovery and may avoid the additional dissection and microsurgical time required for nerve coaptation.

Aesthetic outcome was strongly influenced by injury mechanism rather than by flap choice. Sharp injuries produced the best scars (mean VSS 1.80), whereas crush/avulsion mechanisms had higher scores (mean VSS 3.71). This supports counseling patients with severe contamination and avulsion forces toward more conservative cosmetic expectations from the outset, and emphasizes the importance of meticulous initial debridement. Donor site morbidity was minimal: 70.6% of donor sites were closed primarily, and the remaining 29.4% (all flaps > 10 cm^2^) healed uneventfully after full-thickness skin grafting, without persistent pain, dysesthesia, or hand grip impairment. This favorable donor site profile compares well with crossfinger and free flap alternatives.

Functional motion was good in the majority of patients. The three patients graded as fair all had concomitant extensor tendon involvement, and one had an associated distal phalanx fracture. This pattern suggests that midterm motion is determined more by the underlying tendon and bone injury than by the flap itself, and underscores the need for early structured hand therapy after splint removal at 14 days.

From a regional perspective, our results add a Vietnamese cohort to a small but growing Southeast Asian experience with this flap. Hariyono et al^17^ recently reported successful application of a related reverse dorsoradial flap from the thumb to cover a finger defect in a single Indonesian patient, supporting its versatility outside its original distal thumb indication. Together, these reports indicate that the reverse dorsoradial digital artery flap can be reproducibly performed with consistent outcomes in centers without routine access to microsurgical free tissue transfer.

This study has several limitations. It is a single-center prospective case series of modest size without a comparison group; thus, observed associations between flap area and sensation, or between wound character and scar quality, should be regarded as hypothesis-generating rather than definitive. In particular, the absence of a comparative arm prevents direct comparison with the FDMA (Foucher or kite) flap or the crossfinger flap, and the present data cannot establish superiority over these alternatives; a prospective multicenter matched-cohort or randomized comparison would be required for that purpose.^11^ Follow-up was limited to three months, which captures early functional and aesthetic outcomes but may underestimate further sensory maturation (which typically continues beyond 6 months), scar remodeling (which proceeds for 12–18 months), and any late donor site complaints; a planned 12-month follow-up of the present cohort is in progress. We did not include objective hand-strength measurements (eg, Jamar dynamometry) or validated patient-reported outcome instruments such as the Quick Disabilities of the Arm, Shoulder, and Hand (QuickDASH) or Michigan Hand Questionnaire because a Jamar dynamometer and culturally adapted Vietnamese-language versions of these instruments were not available at our institution during the study period; their incorporation is planned for the next prospective cohort. Innervated variants and a comparison with the FDMA flap and crossfinger flap in a randomized or matched-cohort design would be informative next steps.

Within these limitations, the present series shows that the reverse homodigital dorsoradial digital artery flap is a reliable, single-stage reconstructive option for thumb soft tissue defects exposing tendon or bone, achieving complete coverage and 100% flap survival, good motion in 82.4%, S3 sensory recovery in 70.6%, low scar burden, minimal donor site morbidity, and universal patient satisfaction at three months. With a constant vascular pedicle, thin and pliable glabrous skin, and immobilization confined to the injured thumb, this flap should be considered a primary option in the reconstructive algorithm for thumb soft tissue defects, particularly in centers without routine access to microsurgical free tissue transfer.

## Conflicts of Interest

No benefits in any form have been received or will be received related directly to this article.

## References

[1] Leversedge F.J. (2008). Anatomy and pathomechanics of the thumb. Hand Clin.

[2] Adani R., Tang J.B., Elliot D. (2022). Soft and tissue repair of the hand and digital reconstruction. J Hand Surg Eur Vol.

[3] Khan M., Hayat W., Khan N., Ullah H., Ali Q., Khan R. (2022). Soft tissue reconstruction of thumb: classification of defects and standardization of treatment. Pol Przegl Chir.

[4] Moschella F., Cordova A. (2006). Reverse homodigital dorsal radial flap of the thumb. Plast Reconstr Surg.

[5] Chang S.C., Chen S.L., Chen T.M., Chuang C.J., Cheng T.Y., Wang H.J. (2004). Sensate first dorsal metacarpal artery flap for resurfacing extensive pulp defects of the thumb. Ann Plast Surg.

[6] Gebhard B., Meissl G. (1995). An extended first dorsal metacarpal artery neurovascular island flap. J Hand Surg Br.

[7] Prakash V., Chawla S. (2004). First dorsal metacarpal artery adipofascial flap for a dorsal defect of the thumb. Plast Reconstr Surg.

[8] Sherif M.M. (1994). First dorsal metacarpal artery flap in hand reconstruction. II. Clinical application. J Hand Surg Br.

[9] Goh E., Kulkarni S., Moura F., Norton S. (2024). Reconstruction of soft-tissue defects of the thumb using reverse-flow homodigital flaps: a systematic review. J Hand Microsurg.

[10] Zhang Q., Li W., Chen Q. (2023). Reverse homodigital dorsoradial flaps for thumb coverage obtained good sensory recovery after a long time follow-up. J Plast Surg Hand Surg.

[11] Zyluk A., Flicinski F. (2023). Outcomes of coverage of soft tissue defects in the thumb with a "kite flap". Handchir Mikrochir Plast Chir.

[12] Libberecht K., Lafaire C., Van Hee R. (2006). Evaluation and functional assessment of flexor tendon repair in the hand. Acta Chir Belg.

[13] Alhazmi B., Alhujayri A., Almeshal O., Aldekhayel S., Alshomer F. (2022). Soft Tissue Reconstruction of the Hand: Loco-Regional and Distant Flaps Selection and Approach.

[14] Moschella F., Cordova A., Pirrello R., Brunelli F. (1996). Anatomic basis for the dorsal radial flap of the thumb: clinical applications. Surg Radiol Anat.

[15] Qin H., Ma T., Xia J., Zhang W. (2020). Comparison of reverse dorsoradial flap for thumb reconstruction: narrow pedicle versus wide pedicle. Arch Orthop Trauma Surg.

[16] Bao Q.Y., Xiao C.W., Peng F., Han D., Wang T., Gu Y.D. (2014). Restoration of thumb sensibility with innervated reverse homodigital dorsoradial flap. J Reconstr Microsurg.

[17] Hariyono D.C., Wiramur A., Sungkar A., Yarso K.Y., Philo R. (2023). Reverse dorsoradial metacarpal artery flap for reconstruction of large finger skin defect: a case report. Int J Surg Case Rep.

